# ODG: Omics database generator - a tool for generating, querying, and analyzing multi-omics comparative databases to facilitate biological understanding

**DOI:** 10.1186/s12859-017-1777-7

**Published:** 2017-08-10

**Authors:** Joseph Guhlin, Kevin A. T. Silverstein, Peng Zhou, Peter Tiffin, Nevin D. Young

**Affiliations:** 1 Department of Plant and Microbial Biology, 140 Gortner Laboratory, 1479 Gortner Avenue, University of Minnesota, St. Paul, MN 55108 USA; 2 Minnesota Supercomputing Institute, 599 Walter Library, 117 Pleasant St. SE, Minneapolis, MN 55455 USA; 3 Department of Plant Pathology, 495 Borlaug Hall, 1991 Upper Buford Circle, St. Paul, MN 55108 USA

**Keywords:** Comparative genomics, Non-model species, Annotation, Graph database, Data integration

## Abstract

**Background:**

Rapid generation of omics data in recent years have resulted in vast amounts of disconnected datasets without systemic integration and knowledge building, while individual groups have made customized, annotated datasets available on the web with few ways to link them to in-lab datasets. With so many research groups generating their own data, the ability to relate it to the larger genomic and comparative genomic context is becoming increasingly crucial to make full use of the data.

**Results:**

The Omics Database Generator (ODG) allows users to create customized databases that utilize published genomics data integrated with experimental data which can be queried using a flexible graph database. When provided with omics and experimental data, ODG will create a comparative, multi-dimensional graph database. ODG can import definitions and annotations from other sources such as InterProScan, the Gene Ontology, ENZYME, UniPathway, and others. This annotation data can be especially useful for studying new or understudied species for which transcripts have only been predicted, and rapidly give additional layers of annotation to predicted genes. In better studied species, ODG can perform syntenic annotation translations or rapidly identify characteristics of a set of genes or nucleotide locations, such as hits from an association study. ODG provides a web-based user-interface for configuring the data import and for querying the database. Queries can also be run from the command-line and the database can be queried directly through programming language hooks available for most languages. ODG supports most common genomic formats as well as generic, easy to use tab-separated value format for user-provided annotations.

**Conclusions:**

ODG is a user-friendly database generation and query tool that adapts to the supplied data to produce a comparative genomic database or multi-layered annotation database. ODG provides rapid comparative genomic annotation and is therefore particularly useful for non-model or understudied species. For species for which more data are available, ODG can be used to conduct complex multi-omics, pattern-matching queries.

**Electronic supplementary material:**

The online version of this article (doi:10.1186/s12859-017-1777-7) contains supplementary material, which is available to authorized users.

## Background

Collecting genomic and transcriptomic data has become fast and easy. Making biological sense of these data remains challenging. For model systems curated databases (e.g. MaizeGDB, SoyBase, WormBase) provide powerful tools that integrate, analyze, and visually display complementary data types such as annotated metabolic pathways, characterized genes, and diversity analyses. These curated databases greatly facilitate biological insights [1–4]. Even these curated databases, however, have limitations that impede biologists ability to leverage omics data: complicated multi-omics queries are difficult or impossible due to limits in the underlying database functionality, users are often unable to integrate their own data into the databases, there is little flexibility for users to design specific queries, and many curated datasets, such as pathway or transcriptomic datasets, are limited by the lack of additional integrated annotations and comparative genomics. Despite these limitations, the resources available in curated reference databases available for model systems greatly exceed what is available for non-model systems.

We developed the Omics Database Generator (ODG), an easy to configure database that is amenable for custom installations and use with non-model systems. ODG allows users to integrate and query many data types (genomic, transcriptomic, comparative, variant, ontological, annotated pathway, interactions, etc.) in fast and powerful ways and can easily incorporate new data. ODG also has built in functions for translating annotation between different versions of assembly and annotation. Finally, ODG provides an easy-to-use web interface and command-line to perform queries while the structure of the database allows for flexible queries that would be difficult without extensive modification in most other database platforms. ODG can supplement large-scale curated databases but is flexible enough for individual labs to run on a local machine.

ODG has several advantages over many commonly used genomic databases: i) Advanced query capabilities allow users to explore relationships and ask queries that are virtually impossible with relational databases, and difficult without extensive customization with BioMart-type setups, ii) ODG is easy to setup for any organism and its related species, and automatically generates a query interface, and iii) easy and automatic cross-referencing of gene names and coordinates from one genome release to another (e.g., v 3 to v 5) and from one genome to related species’ genomes. Research and annotation of non-model systems can therefore be facilitated by comparison to model systems to enrich annotation. iv) By using scalable open-source technologies, researchers can run a customized local copy for their lab, and curators of larger database sites can integrate features of ODG into their existing platforms.

## Implementation

### Architecture and importing data

The underlying structure of ODG relies on graph database storage. ODG stores and queries data using Neo4J, an open-source graph database that has recently grown in popularity and maturity [5]. Graph databases differ from relational databases used for most biological databases in that they do not require the data be fit into the underlying database paradigm, something that can be difficult to achieve because many types of biological data do not fit into strict schemas. For example, CHADO, a relational database that underlies many commonly used genomic databases, requires that biological data be fit into strict schema for SQL queries [6]. The result is a data structure unrepresentative of the underlying biological paradigms, and the SQL schema must be learned in order to conduct queries. In SQL relationships between data are typically stored in a separate table, one additional table for each relationship type. Therefore, to maximize the potential of CHADO one must learn both SQL and the CHADO schema itself.

**Fig. 1:**
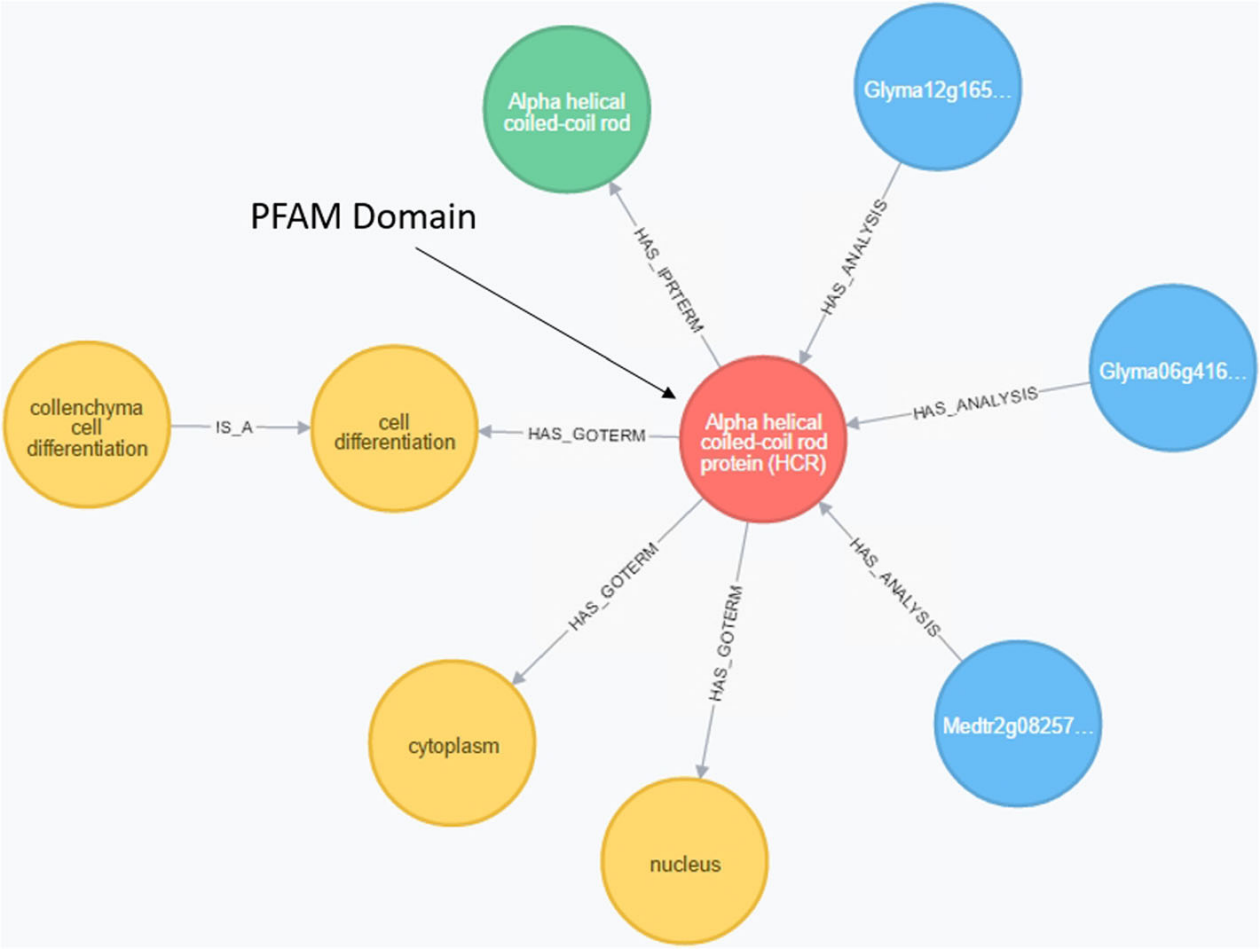
Example of the internal structure of ODG as represented by Neo4J. Here we can see a PFAM domain (red) that has been identified in 2 *Glycine max* genes (Glyma…) and 1 *Medicago truncatula* gene (Medtr…). We can see that this PFAM domain is associated with the GO Terms, represented in yellow, cell differentiation, cytoplasm, and nucleus. The GO Term collenchyma cell differentiation is also a cell differentiation GO term, as determined from the imported definitions from the Gene Ontology consortium. Because of the relationships ODG is able to assign additional annotation to these genes based on a known protein domain family. The query was initiated by looking for genes which may be associated with collenchyma cell differentiation

In contrast, graph databases model the data and their relationships more intuitively and allow for flexible data structures of both nodes, representing data, and edges, representing the relationship between nodes (Fig. 1 *)*. This flexible schema allows for custom fields such as additional annotation and metadata. The stored relationships make the data amenable to easier and more powerful queries. All data are stored locally, making it is possible for users to select genomes and experimental data to include in the database, utilize internally generated data, and prioritize integration of data that are most relevant to specific research projects. This structure allows for flexible queries that would be difficult without extensive modification in other database platforms.

**Fig. 2:**
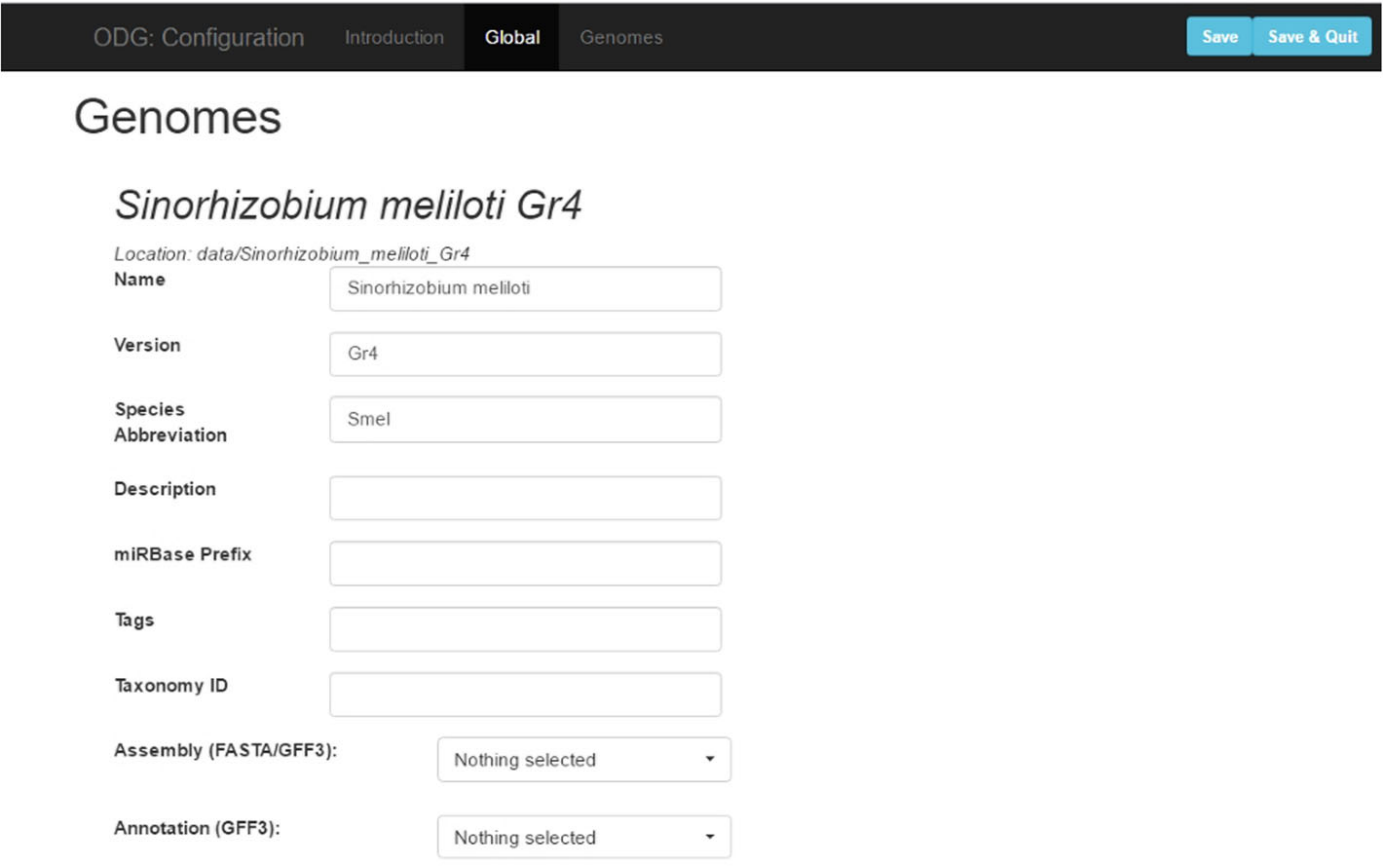
ODG provides a simple web-based configuration utility that uses algorithms to attempt to identify file types and pre-populate many fields

Initial configuration of databases is typically a complex process that requires knowledge and expertise in systems administration. By contrast, initial configuration of ODG is accomplished easily using a web-based tool that leads users through the process and can be installed on a local workstation or laptop with only a basic knowledge of UNIX (Fig. 2). ODG assists in initial configuration and setup by providing necessary scripts and automatically generating a full network-oriented query interface based on input files.

Once ODG is installed and configured, the next step is to perform initial processing steps to identify initial annotation information (i.e., running BLAST+ or InterProScan, if not previously completed) [7, 8]. ODG then imports data specified in the configuration file, including the importing of several omic data types (genomic, transcriptomic, comparative, variant, ontological, annotated pathways, and gene interactions, etc.) with the only limitation being that the data are available in standard file formats (e.g. GFF3, FASTA, TSV, OBO, etc.). New data and resources can be added at any time and ODG can be fully regenerated as needed. ODG saves configurations between uses to facilitate adding or updating data sources.

### Data types and database generation

**Fig. 3:**
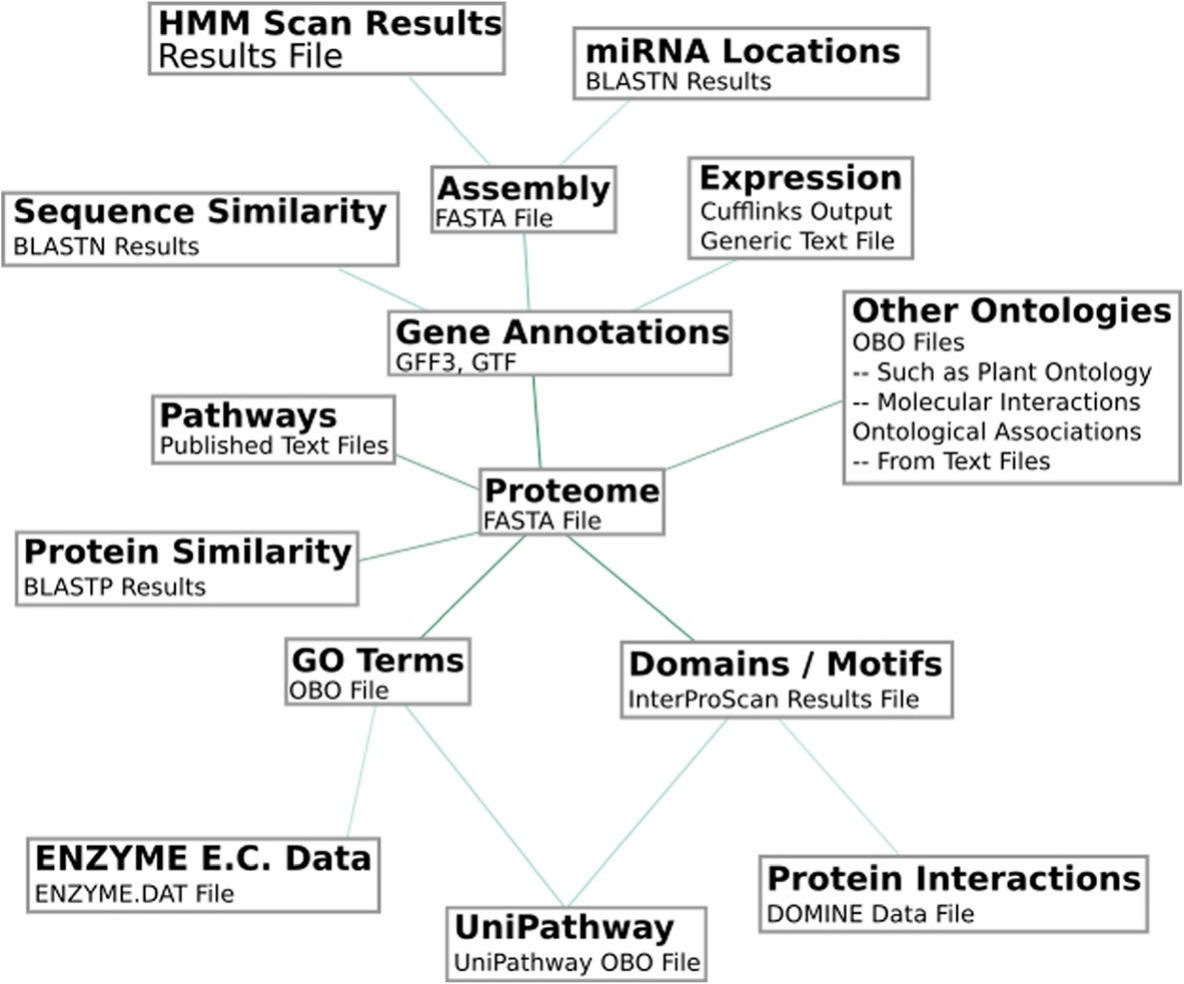
Database dependency structure of ODG. Each data type is further annotated by those connected directly in the graph. For example, a proteome can be linked to UniPathway entries if InterProScan results are present. If both are present, then both can be queried. If all dependencies are present from “HMM Scan Results” to “UniPathway” then it becomes possible to query HMM Scan Results locations and identify nearby genes or proteins and if they have any domains or motifs linking them to UniPathway annotations

ODG will import and create appropriate relationships from annotation files, assemblies, BLAST results, miRBase, Cufflinks expression data, InterProScan results, including motifs, Pfam, Gene3D, and Coils [8–10]. In order to provide further annotation information, the Gene Ontology and ExPASY ENZYME definition files can be imported to add additional data to GO (Gene Ontology) term IDs, which receive relationships from InterProScan results [11]. Pathways from PlantCyc can also imported, but any pathways with the same file format will work [12]. Molecular interactions from BioGRID can also be imported [13]. Other data can be converted into generic file formats to import new attributes or relationships into ODG. Each additional data type imported can add a new dimension of annotation to the database, as illustrated in Fig. 3.

Static data, such as annotations and assemblies, must be obtained from public repositories or provided by researchers. InterProScan and cufflinks must be performed manually by user, due to the complexity and needs for each genome, but common output formats from both programs can be imported directly into ODG. Further guidelines are provided in the user’s guide. Other data must be generated locally, including BLASTP matches and BLASTN matches such as hairpin miRNAs and genomic regions. ODG generates scripts to run BLAST+ programs to ensure compatible output files are created and file names are as expected by the import step. When cufflinks expression files are included in the configuration, ODG will store and record expression conditions, FPKMs, and generate co-expression correlations and store them as relationships.

ODG is well suited for transferring information from one or more model organisms or other well-studied species to a newly sequenced or understudied species. With a minimum of genome sequence and predicted coding sequences it is possible to BLAST these sequences to other organisms and ODG will identify the best reciprocal hits and best syntenic hits. Data from other organisms such as known pathways or expression data will then be associated with these coding sequences. InterProScan can be run to identify known motifs and domains to associate putative genes to GO categories, PFAM domains, and UniPathway annotations which can then be used as a basis for further queries [8]. Additional data such as expression information can provide gene expression patterns and be used in queries.

### Inferred network relationships

Data in ODG is a network based on relationships between data nodes. ODG can work with subnetworks to identify biological patterns. Because not all species have defined co-expression or pathway networks, ODG has the ability to infer network relationships when direct relationships are not available. For example, when an unannotated gene has a top BLASTP hit to an annotated gene that has been placed in the same co-expression network, then the information from the annotated gene can be assayed to provide possible annotations for the unannotated gene, such as GO terms or ENZYME pathway data. The power to infer or transfer annotation information can be particularly useful when working on un-curated datasets or understudied species.

### Annotation translation between genome versions

**Fig. 4:**
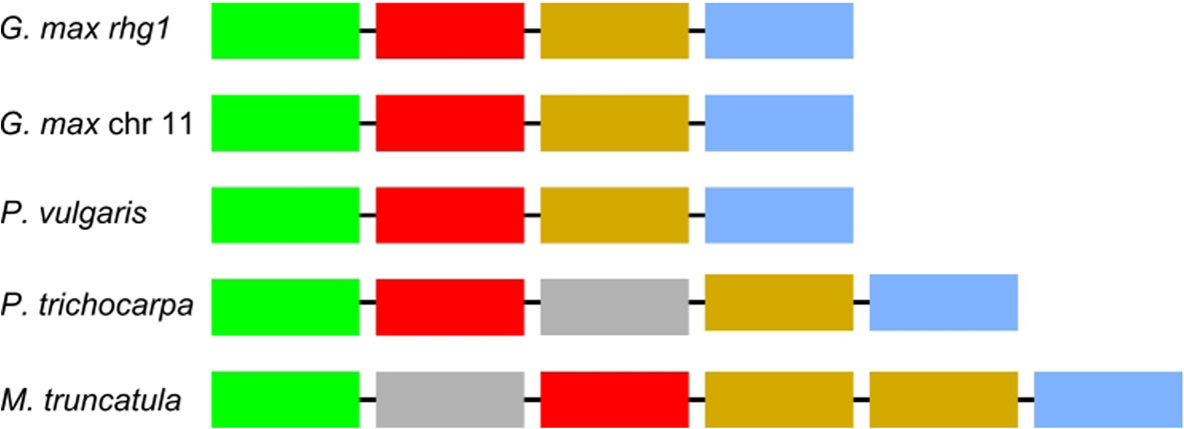
Flexible queries allow searching for syntenic regions across species while allowing for gene deletions or insertions. These are the results of a query against the *rhg1* soybean locus found on chromosome 18. Another locus of similar genes and order is identified on chromosome 11, as well as in other species. In *P. trichocarpa* and *M. truncatula* an unrelated gene is identified breaking up the synteny. In *M. truncatula* there is also a copy of the third gene (orange), which does not break the queries ability to identify the closest syntenic and BLASTP matching region

Updates of genome assemblies and annotations yield versions that are closer to the true representation of genomes, but can cause confusion because different genome versions often differ in gene models, locations, and construction. Currently, annotation between genome versions is accomplished using liftOver which converts coordinates from a previous assembly to coordinates in a new assembly [14]. The liftOver approach is not ideal because it does not account for updated gene models, gene splits, or gene merges. ODG compares annotation versions or transfer annotations by performing an all-vs-all BLASTN to identify gene ID changes, and can detect and mark most gene splits and merges between annotation versions. When confronted with ambiguity, such as multiple top BLAST hits, neighboring genes will be used to identify the correct gene model in both versions by taking into account genic synteny. Identical BLAST hits can be an issue with recent duplications or gene families. The nature of the graph database makes the use of neighboring gene models a flexible query, where changes in the immediate region, such as newly annotated genes or deleted gene models, will not hinder the syntenic search (Fig. 4).

### Performing database queries

**Fig. 5:**
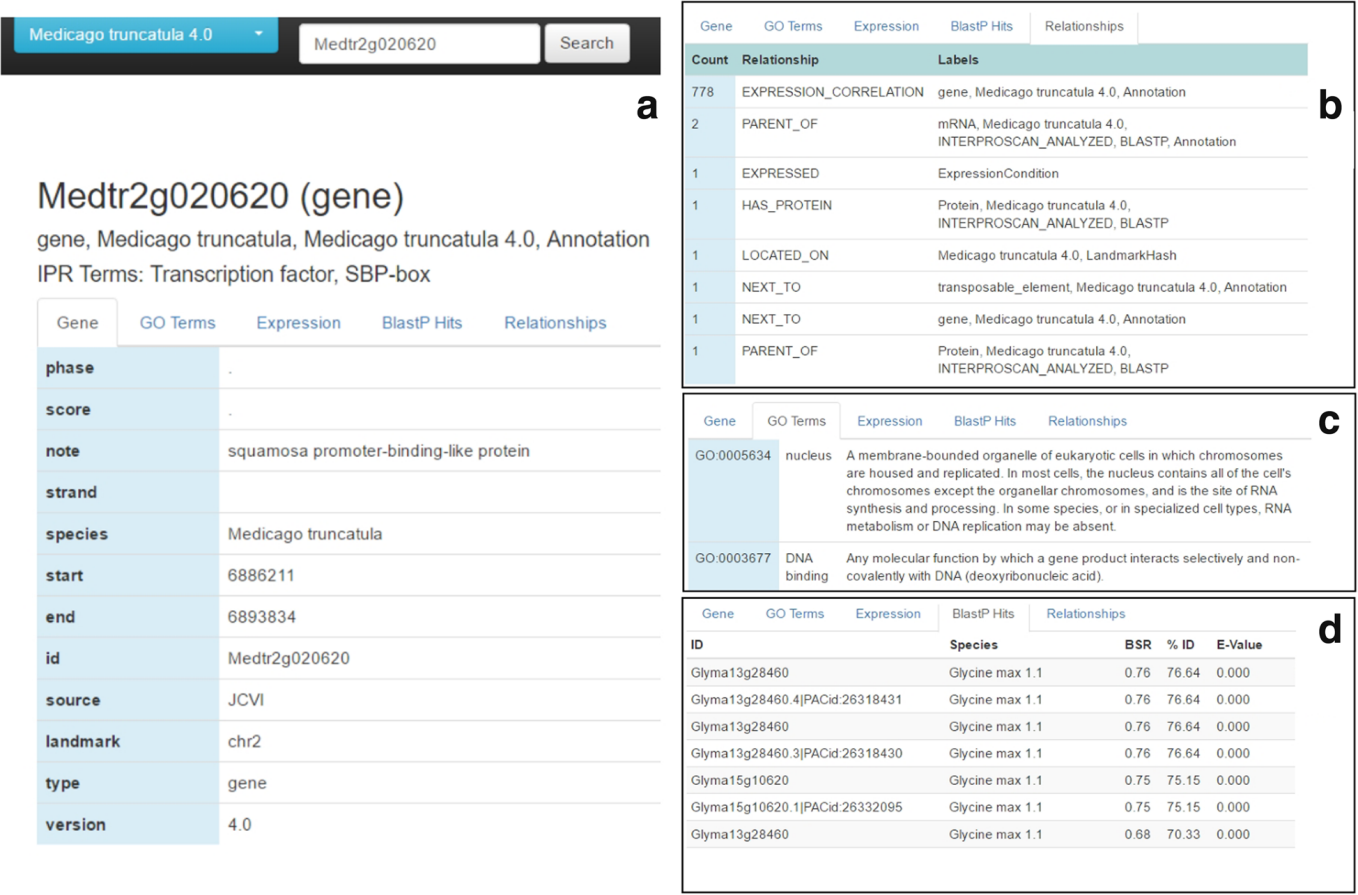
ODG generates a query interface using a web-based interface. **a**) This is the gene-level detail, primarily populated by gene definition entries as well as the IPR Terms, when available. **b**) Summarized here are the relationships attached to this gene node, and the labels of the nodes the relationships connect to. **c**) Gene Ontology (GO) terms that were identified for this gene from InterProScan. **d**) A summary of the BlastP hits for this gene’s predicted protein sequence, including to other species. Provided are the BLAST Score Ratio (BSR), percent identity, and the e-value output from the BLAST+ program

Once data have been imported and the database generated, there are four methods that can be used to query the database. Table 1 lists queries that are built-in to the database. An ODG web query interface can be started with the command: *odg.sh start* and the user can open their browser to http://localhost:6789 to access ODG’s built-in query interface. Figure 5 provides some screen captures from this interface, including additional layers of annotation for the specified gene. For large queries, it is often best to use the command-line. More advanced users can mount the ODG database into the Neo4J query framework and write their own queries, or query directly from a programming language such as R or Java using existing Neo4J adapters and libraries.

Through the web interface and command-line interface (CLI) the database can be queried using individual Gene IDs, a gene list, a set of genomic coordinates, or other feature names, simply by choosing the species to be queried from the drop-down menu and then typing an identifier. ODG will return the node corresponding to the query, its information, and a set of relationships. Each node and relationship may be clicked on to view additional data.

**Table 1:** A list of built-in queries in the database

Built-in Queries	Notes
Co-expression Network	Based on direct or inferred expression
GO Term / Pathway Enrichment	Given a list of genes
Identify nearest genes to SNP markers	Given a set of coordinates
Biological Processes	Given a list of genes
Identify Orthologs	For given genes, for all species in the database
Expression Data	For a given set of genes
GWAS Annotation	Annotate a set of SNPs with nearest gene, genic or not, expression patterns, GO categories and EC categories, plus additional data.
Annotation Translation	Given a list of genes and two or more genome versions of an annotation. Anchored BACs can also facilitate translation.

While ODG has several built-in queries, advanced users can query the database directly using CYPHER, the query language of Neo4j, directly through Neo4J’s web interface or via language hooks available for many programming languages. An example of this is featured in Additional file 1: Fig. S1. ODG also supports many queries via the command-line interface, allowing for larger queries to be executed without the overhead of a web server running in the background. For example, we have used customized command-line queries to pull out useful data from several thousand SNPs identified via GWAS, identify orthologs for a list of genes, determine ENZYME E.C. numbers from GO categories, and to retrieve GO biological process terms for a list of genes. Additional queries may be written in Java or other programming languages.

## Comparative Omics study

To illustrate the usefulness of ODG we examined four recently sequenced and published strains of Rhizobia, a bacterial species which can form nitrogen-fixing nodules with some legumes, a useful trait in agriculture. We examined *Rhizobium aegypticaum sv. trifolii*, *Rhizobium bangladeshense sv. trifolii*, two species with limited foundational research published, and compared these to the well-studied bacteria *Escherichia coli* and *Ensifer meliloti* (previously known as *Sinorhizobium meliloti*), a model bacterium for studying legume-rhizobia symbiosis [15–18]. *R. aegypticaum* and *R. bangladeshense* were isolated from berseem clover in Egypt. *R. aegypticaum*, strain Rhiz950, is salt-tolerant; the three strains of *R. bangladeshense*, Rhiz1002, Rhiz1017, and Rhiz1024, are thermal and pH-tolerant. Genes were predicted for the four rhizobia strains using prodigal [19]. Published annotations of *E. coli* and *E. meliloti* were used in this study. All were compared with BLAST+ using scripts generated by ODG and analyzed with InterProScan 5.24–63.0 [7, 20].

To examine potential causative genes for the salt-tolerance trait in Rhiz950, we compared GO biological process categories of the predicted Rhiz950 proteins to those in the other four strains. In Rhiz950 we identified three genes with GO biological process categories “sodium ion transport” and “oxidation-reduction process” but none in any of the other strains. An additional gene was found related to “sodium ion transport” and “alanine transport” with no orthologs identified in *R. bangladeshense*. Additionally, two more with the combined categories of “sodium ion transport” and “regulation of pH” had two copies in Rhiz950, missing in Rhiz1002, and one copy in the remaining strains (Additional file 2: Table S2).

We further examined the pathways present in all Rhiz950 genes that did not have orthologs in any of the other species (Rhiz950 vs. all). We identified genes belonging to the mevalonate pathway I and isoprene biosynthesis II, as defined by MetaCyc and KEGG [21, 22]. The identified PFAM domains of all genes in Rhiz950 are included as Additional file 2: Table S2.

The *R. bangladeshense* strains contain two genes related to the queuosine biosynthesis pathway not found in any of the other strains, including Rhiz950. Examining PFAM domains reveals 1865 novel genes in these three strains that are related to ABC transporters, 912 belonging to the lysR regulatory family, and 874 having a LysR substrate binding domain. Additional ones are reported in Additional file 2: Table S2.

Additional file 3: Table S3 contains predicted functional annotation derived from the better studied bacteria *E. meliloti* or *E. coli* based on best orthologs. Future directions for this study would likely include transcriptomic data from all species, potential knockouts, and literature searches for existing gene orthologs in either *E. coli* or *E*. meliloti potentially related to these traits.

Using ODG we were able to rapidly identify genes potentially related to the salt-tolerance trait for follow-up studies, identify potential pathways found in Rhiz950, and examine some differences between *R. bangladeshense* strains and the other strains.

## Results and discussion

ODG provides an alternative way of storing and analyzing biological data, brings the ability to host and run custom tailored databases to individual labs and researchers, and provides a platform for connecting newly sequenced or understudied species with annotated species and other genomic and experimental information. By focusing on user-provided data while interacting with existing and published data researchers are able to customize databases and queries for their projects. ODG’s flexibility allows researchers to focus on the data that is important to them, while the interface lowers the computational skills required to build and query the database. ODG could also benefit a larger database warehousing site for a model organism that chose to use its API or query the final database product directly.

ODG builds networks from user-specified input data that provide the basis for all queries. The database has been primarily tested on plants and bacteria, but by working with standard file formats and allowing for some deviation from those standards, should work on a variety of organisms with zero or minimal additional processing. By also accepting user-defined data, the database can be made to fit many research needs.

As demonstrated in the omics study presented above, ODG has the capability to rapidly facilitate annotation, comparative searches, and predicted protein analyses across multiple genomes adding additional dimensions to relatively sparse data. This will accommodate researchers studying new species and well-studied species at the single-gene scale and genomic scales by centralizing much of their data. By allowing additional data-types and being built using within an open-source database engine, ODG enables researchers to add in new layers of data which can then be made available to the lab or potentially to the public.

## Conclusions

ODG changes how researchers store and query data at genome-scales, facilitating knowledge transfer from prior work on model organisms and related species. ODG is most useful to researchers working with understudied organisms, as well as those performing genome-wide studies where many loci are queried at once. Full usage of ODG requires database generation, allowing users to utilize appropriate data sources and types for their project. Once database generation is complete, a query mode is available, allowing usage by a web browser for basic queries yet providing a programmatic interface for more advanced users.

## Availability and requirements

**Project name:** Omics Database Generator

**Project home page:** https://github.com/jguhlin/odg

**Operating system(s):** Mac OS X, Windows, Linux, Unix

**Programming language:** Clojure, Java

**Other requirements:** Java 1.8 or higher

**License:** GNU GPL v3

**Any restrictions to use by non-academics:** None

## Additional files

Additional file 1: Figure S1. Advanced users can query ODG using Neo4j’s query language CYPHER. Presented is an example identifying HMM Matches to nearby genes and aggregating GO term counts, requiring GO terms to be labelled as a biological process. (JPEG 274 kb)

Additional file 2: Table S2. PFam Domains and biological process GO categories for the four rhizobia strains. Predicted proteins related to multiple GO biological process categories are joined together with the pipe character. (XLSX 639 kb)

Additional file 3: Table S3. Gene annotations of top scoring BLAST+ hits for the predicted genes in the four rhizobia strains, as inferred from *E. coli MG1655* and *E. meliloti* 1021. (XLSX 383 kb)

## Abbreviations

GO: Gene Ontology
ODG: Omics Database Generator
