# Supplementary figures and images for: ODG: Omics database generator - a tool for generating, querying, and analyzing multi-omics comparative databases to facilitate biological understanding
Source: BMC Bioinformatics. 2017 Aug 10;18:367. doi: 10.1186/s12859-017-1777-7 (PMC5553995; doi:10.1186/s12859-017-1777-7)

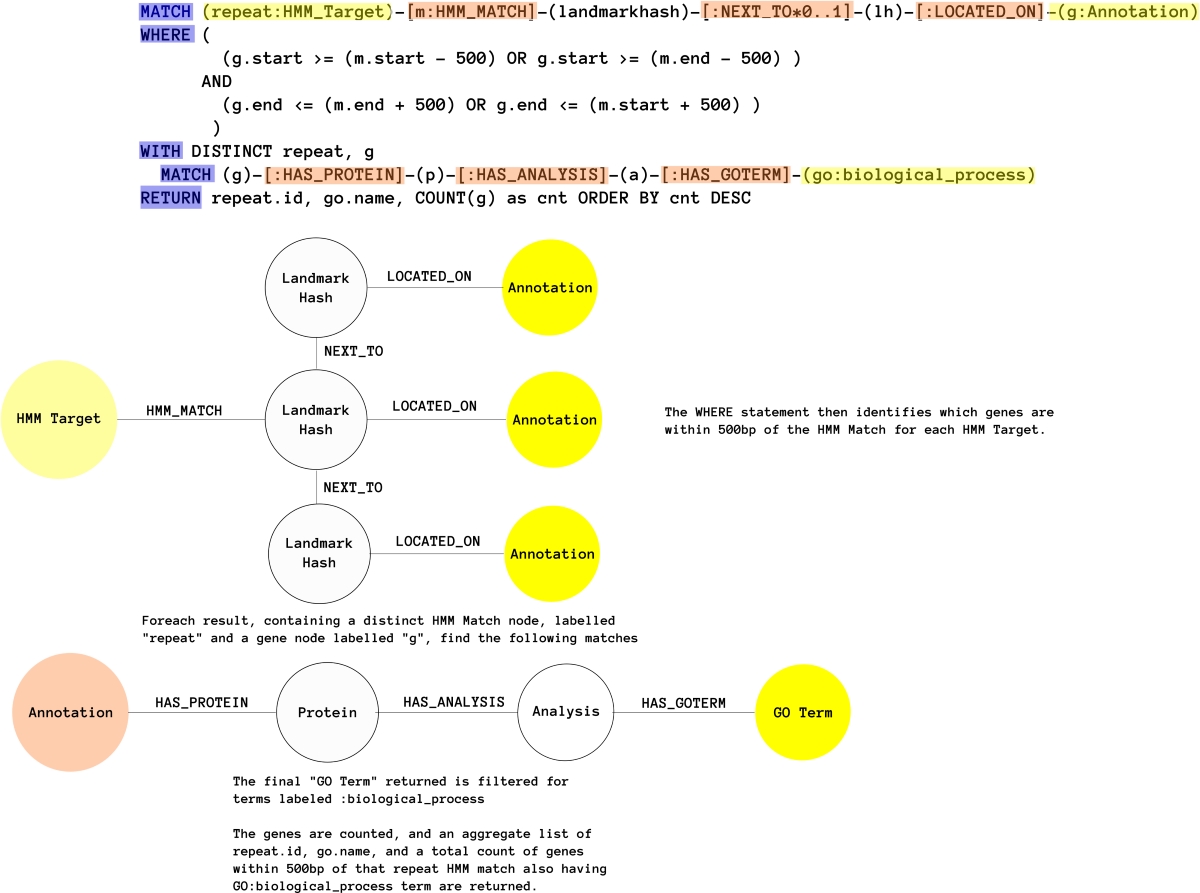

Supplement: Supplementary file 1 — Advanced users can query ODG using Neo4j’s query language CYPHER. Presented is an example identifying HMM Matches to nearby genes and aggregating GO term counts, requiring GO terms to be labelled as a biological process. (JPEG 274 kb) [file 12859_2017_1777_MOESM1_ESM.jpg]
